# Remote Assessments of Hand Function in Neurological Disorders: Systematic Review

**DOI:** 10.2196/33157

**Published:** 2022-03-09

**Authors:** Arpita Gopal, Wan-Yu Hsu, Diane D Allen, Riley Bove

**Affiliations:** 1 Weill Institute of Neurosciences University of California San Francisco San Francisco, CA United States; 2 Department of Physical Therapy and Rehabilitation Science University of California San Francisco/San Francisco State University San Francisco, CA United States

**Keywords:** neurological disease, hand function, remote assessment, assessment, telemedicine, rehabilitation, telerehabilitation, review, neurological, hand, function, diagnosis, intervention, dysfunction, feasibility, mobile phone

## Abstract

**Background:**

Loss of fine motor skills is observed in many neurological diseases, and remote monitoring assessments can aid in early diagnosis and intervention. Hand function can be regularly assessed to monitor loss of fine motor skills in people with central nervous system disorders; however, there are challenges to in-clinic assessments. Remotely assessing hand function could facilitate monitoring and supporting of early diagnosis and intervention when warranted.

**Objective:**

Remote assessments can facilitate the tracking of limitations, aiding in early diagnosis and intervention. This study aims to systematically review existing evidence regarding the remote assessment of hand function in populations with chronic neurological dysfunction.

**Methods:**

PubMed and MEDLINE, CINAHL, Web of Science, and Embase were searched for studies that reported remote assessment of hand function (ie, outside of traditional in-person clinical settings) in adults with chronic central nervous system disorders. We excluded studies that included participants with orthopedic upper limb dysfunction or used tools for intervention and treatment. We extracted data on the evaluated hand function domains, validity and reliability, feasibility, and stage of development.

**Results:**

In total, 74 studies met the inclusion criteria for Parkinson disease (n=57, 77% studies), stroke (n=9, 12%), multiple sclerosis (n=6, 8%), spinal cord injury (n=1, 1%), and amyotrophic lateral sclerosis (n=1, 1%). Three assessment modalities were identified: external device (eg, wrist-worn accelerometer), smartphone or tablet, and telerehabilitation. The feasibility and overall participant acceptability were high. The most common hand function domains assessed included finger tapping speed (fine motor control and rigidity), hand tremor (pharmacological and rehabilitation efficacy), and finger dexterity (manipulation of small objects required for daily tasks) and handwriting (coordination). Although validity and reliability data were heterogeneous across studies, statistically significant correlations with traditional in-clinic metrics were most commonly reported for telerehabilitation and smartphone or tablet apps. The most readily implementable assessments were smartphone or tablet-based.

**Conclusions:**

The findings show that remote assessment of hand function is feasible in neurological disorders. Although varied, the assessments allow clinicians to objectively record performance in multiple hand function domains, improving the reliability of traditional in-clinic assessments. Remote assessments, particularly via telerehabilitation and smartphone- or tablet-based apps that align with in-clinic metrics, facilitate clinic to home transitions, have few barriers to implementation, and prompt remote identification and treatment of hand function impairments.

## Introduction

### Background

Normally functioning human hands allow everyday participation in self-care, work, and leisure activities that involve precise grip and object manipulation [1]. Specifically, daily activities and fine motor tasks require finger dexterity, thumb-finger opposition, and hand opening-closing, which adapt to task requirements, including those needed to navigate the *digital world*. [2] Unfortunately, chronic disorders of the central nervous system (CNS) can impair hand function even during the early stages of the disease [3]. Damage to the CNS, including the spinal cord, can result in tremor, spasticity, sensory loss, weakness, and coordination loss in the upper limbs, which can negatively impact the ability to adapt to task requirements, thus limiting independence in activities of daily living (ADL) and quality of life [3]. For example, most individuals with Parkinson disease (PD) develop hand tremors over the course of the disorder, leading to difficulty with precise finger and hand movements [4]. In addition, ischemic strokes occur most commonly in the cortical regions supplied by the middle cerebral artery [5], affecting areas of the motor and sensory cortices responsible for the fine motor activity of the hands [6]. In these disorders and others, evaluating hand function at regular intervals can detect changes signaling neurological decline, or monitor response to disease-modifying therapies, symptomatic therapies, or rehabilitation.

Although assessments of hand function are routinely performed in clinics, clinicians have an increasing interest in deploying tools to measure hand function remotely. In-home remote monitoring of function, in general, provides benefits to patients by increasing convenience, reducing travel, and providing the ability to capture data more frequently. Over the past decade, many studies have examined remote monitoring devices in neurological and nonneurological populations [7,8]. For example, in multiple sclerosis (MS), studies have shown that continuous remote monitoring of ambulatory step count can capture—and even predict—changes in MS-related disability and can serve as a longitudinal outcome measure for targeted interventions [9,10]. To date, reviews have mainly focused on lower extremity function or overall physical activity [11]; in fact, the methodological discrepancies in remote device use and reporting regarding hand function have yielded conflicting results in terms of validity, reliability, and ease of clinical use.

### Objectives

In this systematic review, we evaluate the existing evidence regarding remote assessment devices for hand function in populations with chronic CNS disorders. We specifically examine evidence of validity, reliability, and feasibility for each domain of hand function and the stage of development of the assessments. Our findings are expected to facilitate ready implementation of remote assessment of hand function in prevalent neurological disorders.

## Methods

### Eligibility Criteria

This review was structured using the PRISMA (Preferred Reporting Items for Systematic Reviews and Meta-Analyses) [12] framework. Studies were included based on the following criteria: (1) participants had chronic neurological pathologies of the CNS, (2) participants were aged ≥18 years, (3) the studies were peer reviewed and original, (4) the studies were designed to objectively assess hand function, and (5) the assessments were deployable remotely (ie, outside of traditional in-person clinical settings). Studies were excluded if they were (1) conducted in participants with orthopedic impairments of the wrist or hand, (2) conducted in nonhuman primates, (3) designed as an intervention to improve an aspect of hand function (as the intent was to focus on assessment tools rather than a change of function), or (4) not published in English.

### Search Procedures

A literature search was performed using the following databases: PubMed and MEDLINE, CINAHL, Web of Science, and Embase. The search was conducted using both Medical Subject Heading terms and the following keywords independently and in combination: *remote*, *assessment*, *outcome*, *test*, *measurement*, *hand*, *upper extremity*, *arm*, and *function*. Independently, 2 researchers (AG and WYH) assessed articles for relevance and adherence to the eligibility criteria. Studies were recursively searched to identify cited and cited-by articles.

### Data Extraction and Categorization

To evaluate the methodological quality of the included studies, we used the National Institutes of Health quality assessment for observational cohort and cross-sectional studies [13]. Each study was evaluated according to 8 criteria. The overall study quality was assessed as *good* (>5 criteria met), *fair* (4-5 criteria met), or *poor* (<5 criteria met).

The data were extracted (AG) and checked (WYH); discrepancies were resolved through discussion with the senior author (RB). The variables of interest included participant demographics, study design and duration, device type and modality, disease-specific severity levels, comparison assessments, and stage of development and implementation (to understand whether assessments were currently available for use). Participant satisfaction with the study protocol and assessment and time taken to complete the novel assessment were extracted when available. Extracted statistical data included concurrent validity (defined as the comparison between a new test and a well-established one [14]) and reliability (defined as a measure of stability or consistency [15]).

The selected studies evaluated many variables relating to hand function. To compare the most salient domains across studies, we classified assessments into the following hand function domains based on the Functional Repertoire of the Hand established by the American Journal of Occupational Therapy [16]: (1) finger tapping, which is the speed and accuracy of finger taps onto a prespecified target; (2) whole hand grasp, which is the range of motion and coordination of full hand movement; (3) pincer grasp, which is the range of motion and coordination of thumb to index finger movement; (4) hand tremor, which is the quantification of tremor distal to the wrist at rest; (5) reaction time, which is the time taken to respond to a predetermined stimulus using only fingers; (6) pinch and grip strength, which is the quantification of the maximum pinching and gripping strength; (7) finger dexterity, which is the in-hand manipulation of an object; (8) handwriting, which is the clarity and accuracy in drawing or writing; (9) ADL, encompassing tasks required for self-care independence [17]; and (10) instrumental ADL (IADL), encompassing tasks required for household or community-level independence [18].

## Results

### Search Strategy

**Figure 1:**
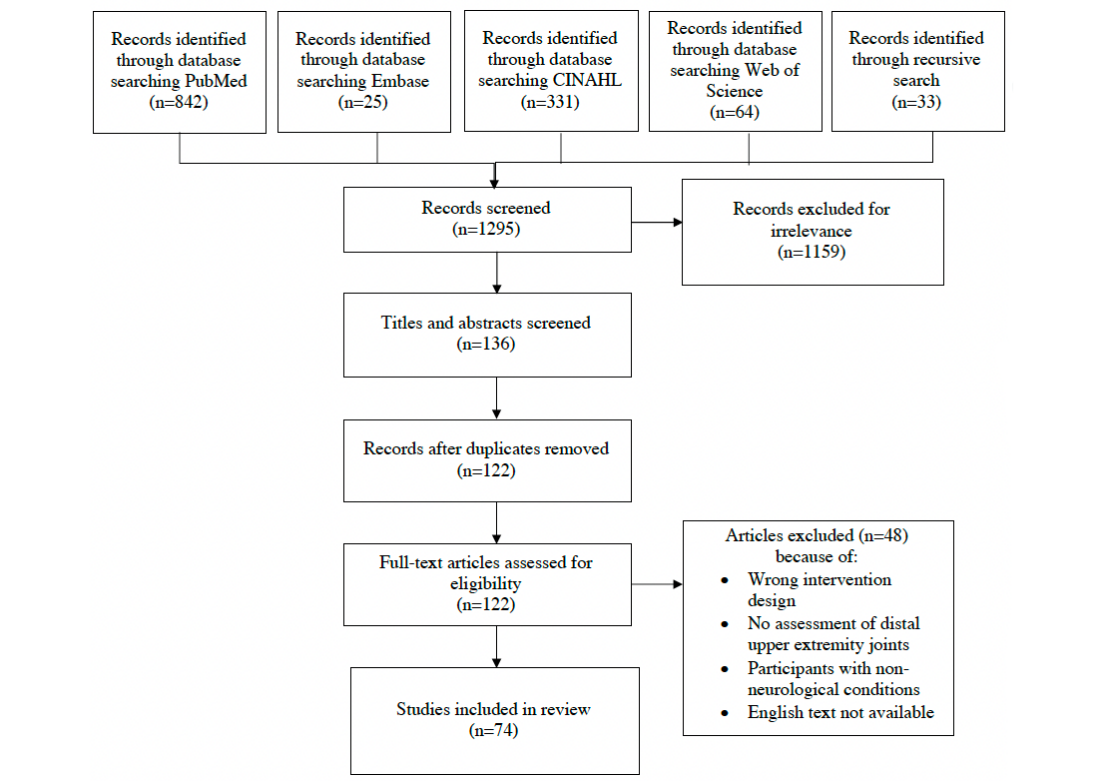
PRISMA (Preferred Reporting Items for Systematic Reviews and Meta-Analyses) diagram outlining study selection.

A search of databases in June 2021 identified 1295 studies, and 33 additional studies were identified through recursive searches. After title and abstract screening and removal of duplicates, 9.42% (122/1295) of studies remained, and the full texts were assessed for eligibility based on the inclusion and exclusion criteria. Approximately 41% (50/122) of full-text studies were excluded for not meeting the inclusion criteria. The final 74 studies were confirmed by a second reviewer (WYH) to have met all eligibility criteria. The PRISMA diagram of the search process is outlined in Figure 1, and individual studies are summarized in Multimedia Appendix 1 [19-90]. Of the 74 studies reviewed, 49 (66%) were rated *good* in terms of overall methodological quality, 14 (19%) were rated *fair*, and 9 (12%) were rated *poor*. Study quality is summarized in Multimedia Appendix 2 [19-90].

### Modalities of Hand Function Assessment

Across the included studies, 3 different modalities of assessment devices were used, summarized in Multimedia Appendix 1. The most frequently used assessment was an external device specific to hand assessment, with the most common types being wrist-worn accelerometers [19-37] and specialized keyboards [38-47]. These designated external devices allowed the collection of information on reaction time, finger tapping speed, and finger dexterity. Although many study authors noted that their external devices were able to capture granular, specific data, many devices were developed under proprietary agreements and are not currently commercially available. The second most common type of assessment was generic smartphone- or tablet-based electronic devices adapted for hand assessment [48-59] or suites of assessments [60-66]. These assessments included an app designed to test finger tapping speed and the accuracy of drawing and tracing various shapes. Such apps facilitated the gathering of data on specific hand function domains at a relatively low cost for people who already had these electronic devices. Finally, 4% (3/74) of studies used telerehabilitation platforms to validate remote administration of well-established in-clinic assessments [67-69]. For example, Amano et al [69] validated the administration of the Fugl-Meyer Assessment and Action Research Arm Test (ARAT) via telehealth platforms, allowing clinical researchers to gather standardized outcome data through secure telehealth tools.

Most of the included studies (51/74, 69%) performed same-day, cross-sectional validation experiments where participants completed novel and comparative assessments at the same time point. However, 28% (21/74) of studies [23,25,36,37,42,46,47,61,63,64,66,70-80] remotely monitored participants’ hand function longitudinally. The duration of the remote monitoring period was 3 days [37] to 3 years [77]. Participant retention and adherence were reported by 5% (4/74) studies [61,66,75,76], all of which had >90% participant retention.

### Target Population

The included studies targeted 5 populations of patients with neurological conditions. Most studies (57/74, 77%) included individuals with PD [37,45,65,67,68,70,73,81]. Other populations evaluated were those with stroke (9/74, 12%) [71,72,82] and MS (6/74, 8%) [59,66,91]. Neurological conditions designated as spinal cord injury [83] and amyotrophic lateral sclerosis [47] were described in 1% (1/74) of studies each.

Most included studies evaluated individuals with mild to moderate disease severity on average, as graded by established disease-specific metrics (eg, the Movement Disorder Society–Unified Parkinson’s Disease Rating Scale [MDS-UPDRS] and the Expanded Disability Status Scale for people with MS) [37,45,59,65,67,69,70,73,82]. Only 8% (6/74) of studies specified the inclusion criteria to limit recruitment to participants with mild to moderate disease severity [37,41,53,58,63,71].

The sample sizes of studies varied between 1 (case study) [26] and 495 participants [66] in the experimental groups. Most studies (41/74, 55%) included control groups of healthy individuals or those with nonneurological conditions in determining the discriminant validity of the assessments (Multimedia Appendix 1).

### Validity and Reliability

Validity data were reported by 73% (54/74) of heterogeneous studies for comparison with well-established in-clinic assessments (Table 1). Approximately 12% (9/74) of studies examining external devices reported high, statistically significant correlations with well-established assessments [19,20,47,50,52,72,73,83,91]. In addition, 8% (6/74) of studies using smartphone assessments [28,49,52,66,79,84] and 1% (1/74) of studies using telerehabilitation [69] found moderate to high, statistically significant correlations with well-established assessments.

**Table 1:** Validity and reliability.

Study	Comparison assessment	Validity	Reliability
Adams [46]	—^a^	Hand tremor (AUC^b^=0.76)	—
Aghanavesi et al [48]	MDS-UPDRS^c^	Finger tapping (r=0.23) Handwriting (r=0.46)	Interrater reliability: Finger tapping (r=0.61) Handwriting (r=0.65)
Akram et al [38]	MDS-UPDRS	Finger tapping (r=−0.49; *P*<.001)	—
Albani et al [73]	MDS-UPDRS	Finger tapping (ICC^d^=0.73)	—
Amano et al [69]	In-clinic assessment	Finger dexterity (r=0.99) Whole hand grasp (r=0.99) Pincer grasp (r=0.99)	Interrater reliability: Finger dexterity (r=0.99)
Arora et al [70]	MDS-UPDRS	Finger tapping (mean error of 1.26 UPDRS^e^ points)	—
Arroyo-Gallego et al [49]	MDS-UPDRS	Finger tapping (AUC=0.85; *P*<.001)	—
Bazgir et al [50]	MDS-UPDRS	Hand tremor (97% accuracy)	—
Bochniewicz et al [82]	ARAT^f^	IADL^g^ (r=−0.14; *P*=.70)	—
Boroojerdi et al [37]	MDS-UPDRS	Finger tapping (r=0.291) Hand tremor (r=0.746)	—
Burdea et al [71]	—	—	—
Cabrera-Martos et al [67]	In-clinic assessment	—	Interrater reliability: Finger dexterity (r=0.89) Finger tapping (r=1.0) Hand tremor (r=0.99)
Cai et al [19]	MDS-UPDRS	Hand tremor (r^2^=0.95)	—
Channa et al [20]	MDS-UPDRS	Hand tremor (91.7% accuracy)	—
Cole et al [21]	MDS-UPDRS	—	—
Creagh et al [59]	9HPT^h^	Handwriting: dominant hand (r^2^=0.39) and nondominant hand (r^2^=0.41)	—
Cunningham et al [74]	—	—	—
Dai et al [22]	MDS-UPDRS	Finger tapping (r=−0.970; *P*<.01) Hand tremor (r=0.93; *P*<.001)	Interrater agreement (Kendall *W*): Finger tapping (0.86) Hand tremor (0.84)
Dubuisson et al [91]	9HPT	Finger dexterity (r=0.9; *P*<.001)	—
Ferreira et al [23]	MDS-UPDRS	—	—
Giancardo et al [39]	MDS-UPDRS	Finger tapping (AUC=0.75)	—
Giuffrida et al [24]	MDS-UPDRS	Hand tremor (r=0.89)	—
Goetz et al [75]	MDS-UPDRS	—	—
Halloran et al [25]	CAHAI^i^	ADL^j^ (r= 0.63; *P*<.001)	—
Heijmans et al [26]	ESM^k^ app (tremor questionnaire)	Hand tremor (r=0.43)	—
Hoffman et al [68]	In-clinic assessment	Hand tremor (83.3% agreement) Handwriting (41.6% agreement)	Interrater reliability: Finger dexterity (r=0.99)
Hssayeni et al [27]	MDS-UPDRS	Hand tremor (r=0.84)	—
Iakovakis et al [52]	MDS-UPDRS	Finger tapping (AUC=0.92)	—
Iakovakis et al [51]	MDS-UPDRS	Finger tapping (r=0.66)	—
Jeon et al [28]	MDS-UPDRS	Hand tremor (85.5% agreement)	—
Jha et al [60]	MDS-UPDRS	Hand tremor (κ=0.68; *P*<.001, substantial) Finger tapping (κ=0.54; *P*<.001, moderate)	Interrater agreement: Hand tremor (96%) Finger tapping (50%)
Kim et al [29]	MDS-UPDRS	Hand tremor (85% accuracy)	Interrater reliability: Hand tremor (r=0.78)
Kleinholdermann et al [85]	MDS-UPDRS	Finger tapping (r=0.445)	—
Kostikis et al [81]	MDS-UPDRS	Hand tremor,: right hand (r=0.75; *P*<.001) and left hand (r=0.85; *P*<.001)	—
Lam et al [41]	9HPT	Finger dexterity (r=−0.553)	Test–retest reliability: Finger dexterity (ICC 0.601)
Lee et al [53]	MDS-UPDRS	Finger tapping (AUC=0.92, 95% CI 0.88-0.96)	—
Lee et al [84]	FMA^l^	Whole hand grasp (92% accuracy)	—
Lee et al [54]	MDS-UPDRS	—	—
Lin et al [88]	—	—	—
Lipsmeier et al [61]	MDS-UPDRS	Finger tapping (*t*=2.18; *P*=.03) Hand tremor (*t*=2.17; *P*=.03)	Test–retest reliability: Finger tapping (ICC=0.64) Hand tremor (ICC=0.90)
Londral et al [47]	—	—	Test–retest reliability: r=0.96; *P*=.09
Lopez-Blanco et al [76]	MDS-UPDRS	Hand tremor (r=0.81; *P*<.001)	Interrater reliability: Hand tremor (ICC=0.89)
Mahadevan et al [30]	MDS-UPDRS	Hand tremor (r=0.67; *P*<.001)	Interrater reliability: Hand tremor (ICC=0.75)
Matarazzo et al [42]	UPDRS-3	—	—
Memedi et al [77]	Visual assessment	Handwriting (85% accuracy)	Test–retest reliability: Handwriting (ICC=0.69)
Mera et al [31]	—	—	—
Mitsi et al [65]	MDS-UPDRS	—	—
Noyce et al [43]	MDS-UPDRS	Finger tapping (r=0.53)	—
Orozco-Arroyave et al [62]	UPDRS-3	—	—
Pan et al [63]	MDS-UPDRS	Hand tremor (r=0.81)	—
Papadopoulos et al [40,55]	MDS-UPDRS	—	—
Powers et al [78]	MDS-UPDRS	Hand tremor (r=0.72)	—
Pratap et al [66]	Longitudinal Neuro-QoL^m^ scores	Finger tapping (β=.40; *P*<.001)	—
Prochazka and Kowalczewski [83]	ARAT and FMA	Finger dexterity (r^2^=0.49) Whole hand grasp, (r^2^=0.88) Pincer grasp (r^2^=0.88)	Test–retest reliability: 0.67% (SD 3.6)
Rigas et al [32]	MDS-UPDRS	Hand tremor (87% accuracy)	—
Salarian et al [87]	MDS-UPDRS	Hand tremor (r=0.87; *P*<.001)	—
San-Segundo et al [33]	—	—	—
Sanchez-Perez et al [34]	MDS-UPDRS	—	—
Schallert et al [56]	—	—	—
Shribman et al [44]	9HPT	Finger tapping (r=0.926)	—
Sigcha et al [79]	MDS-UPDRS	Hand tremor (r=0.969)	—
Simonet et al [57]	MDS-UPDRS	Finger tapping (r=−0.49)	—
Stamatakis et al [35]	MDS-UPDRS	Finger tapping (Goodman–Kruskal index=0.961)	—
Tavares et al [86]	MDS-UPDRS	Finger tapping (r=0.67; *P*<.001)	—
Trager et al [45]	MDS-UPDRS	Finger dexterity (r=0.14; *P*=.43) Finger tapping (r=0.58; *P*<.001)	—
Westin et al [80]	MDS-UPDRS	Handwriting (r=0.41)	Test–retest reliability: Handwriting (r=0.71)
Wissel et al [58]	MDS-UPDRS	Finger tapping (r=0.55)	Test–retest reliability: Finger tapping (r>0.75)
Wu et al [89]	MDS-UPDRS	Hand tremor (r=−0.798)	—
Yu et al [72]	FMA	Finger dexterity (r^2^=0.70) Pinch strength (r^2^=0.72)	—
Zambrana et al [90]	—	—	—
Zhan et al [64]	MDS-UPDRS	Finger tapping (mean 71%, SD 0.4%)	—
Zhang et al [36]	MDS-UPDRS	Hand tremor (85.9% accuracy)	—

^a^Data unavailable.

^b^AUC: area under the curve.

^c^MDS-UPDRS: Movement Disorder Society–Unified Parkinson’s Disease Rating Scale.

^d^ICC: interclass coefficient.

^e^UPDRS: Unified Parkinson’s Disease Rating Scale.

^f^ARAT: Action Research Arm Test.

^g^IADL: instrumental activities of daily living.

^h^9HPT: 9-hole peg test.

^i^CAHAI: Chedoke Arm and Hand Inventory.

^j^ADL: activities of daily living.

^k^ESM: experience sampling method.

^l^FMA: Fugl-Meyer Assessment.

^m^QoL: quality of life.

Of the 74 studies, 15 (20%) heterogeneous studies reported reliability statistics; 2 (3%) telerehabilitation assessments [68,69] revealed a high, statistically significant interrater reliability; and 1 (1%) external device assessment [76] revealed a high, although statistically insignificant reliability.

### Hand Function Domain, Based on the Functional Repertoire of the Hand

#### Finger Tapping Speed

The most common hand function domain assessed was finger tapping speed [22,31,35,37-39,42-45,48,49,51-54,57,58,60-62, 64-67,70,73,75,80,85,86,92]. Finger tapping can provide clinicians with an understanding of fine motor control and stiffness, especially in individuals with spasticity. Of the included studies that examined finger tapping, Albani et al [73] reported the highest correlation with MDS-UPDRS scores in participants with PD. In their study, the authors used an external device, a gesture-based tracking system involving a specialized depth camera and gloves with colored markers, to track and quantify fine hand movements. The MDS-UPDRS item on finger tapping relies on visual assessments of finger tapping (eg, interruptions in the tapping rhythm), and specialized equipment such as an external device aid in quantifying finger tapping capability [73].

#### Hand Tremor

The second most commonly assessed domain was hand tremor, a prevalent impairment in many neurological disorders. Quantifying tremors can help determine the efficacy of pharmacological and rehabilitative therapies. The studies that examined this domain were conducted in participants with PD [19-21,23,24,26-30,32-34,36,37,46,50,55,60,61,63,64,67,68,74-76,78,79,81,87]. Hoffman et al [68] found a 100% agreement of their visual examination of hand tremor at rest in their evaluation of telerehabilitation administration of the MDS-UPDRS assessment in comparison with in-clinic evaluation. Sigcha et al [79] developed a novel smartphone app using an internal gyroscope and accelerometer to measure resting hand tremors. This method had a strong correlation (r=0.97) with in-clinic MDS-UPDRS resting hand tremor scores.

#### Finger Dexterity

The third most commonly assessed domain was finger dexterity [41,45,47,67,68,72,83,88,91]. Finger dexterity assessment tasks included manipulation of small objects (eg, the 9-hole peg test [9HPT] and the coin rotation test), which are useful metrics of fine motor control required for ADL, such as buttoning clothing. Finger dexterity was examined in all 5 of the neurological conditions examined in this review. Of the included studies examining participants with PD, Cabrera-Martos et al [67] found a mean difference of 0.3 (SD 1.2) in scores between telerehabilitation and in-clinic administration of the coin rotation task [93] in the affected limb. Similarly, using telerehabilitation to examine the pinch domain of participants with stroke, Amano et al [69] reported a Spearman ρ of 0.99 between telerehabilitation and in-clinic administered items. In participants with MS, Dubuisson et al [91] validated an external device, a cardboard 9HPT with a correlation of 0.96 between this novel assessment tool and a standard, plastic 9HPT.

#### Handwriting

Approximately 8% (6/74) of studies [48,56,59,68,77,80] examined handwriting accuracy, a specific and sensitive measure of fine motor coordination. The greatest accuracy in comparison with in-clinic assessments was reported by Hoffman et al [68], who found a high percentage of agreement (85%) between in-clinic measures and an external telemetry device of the MDS-UPDRS item for handwriting.

#### Specific Functions

Specific functional domains were evaluated by 11% (8/74) of studies. Grip and pinch strength were examined in 4% (3/74) of studies [68,72,83] using remote deployment of these standard in-clinic metrics. Prochazka et al [83] evaluated the validity of a novel external device to collect force data from grip and pinch tasks and found a coefficient of determination (R^2^) of 0.88 between the remote device and in-clinic administered ARAT. Only 4% (3/74) of studies [25,68,82] specifically examined ADL and IADL. Hoffman et al [68] compared in-clinic and telerehabilitation-administered functional independence measures and found 100% agreement in scores for eating and 91.7% agreement for dressing. Bochniewicz et al [82] developed a wrist-worn accelerometer to capture and quantify disability in individuals after stroke. The protocol simulated IADL such as doing laundry and shopping in a grocery store, and the authors reported 88.4% accuracy compared with ARAT scores of upper extremity functional use.

### Participant Acceptability

In populations with PD, 9% (7/74) of studies reported participant acceptability and usability of assessments. Albani et al [73] found that participants rated the hand gesture–based tracking system 5.9/7 on a poststudy usability questionnaire, indicating ease of use, high interface quality, and usefulness. In 4% (3/74) of studies [24,30,37], participants using wearable sensors to monitor hand tremors and finger tapping found the devices comfortable and easy to use. Both Goetz et al [75] and Ferreira et al [23] reported >80% of participant satisfaction with external devices to examine hand tremors. Mitsi et al [65] found that 76% of participants using a tablet-based assessment for finger tapping [65] and reaction time found it easy to use, with an additional 63% reporting willingness to use it long-term to monitor disease activity.

In populations with stroke, Burdea et al [71] asked both participants and caregivers to provide feedback on their video game–like assessment and intervention using a 5-point study-specific Likert scale (higher scores indicating statement agreement). Participants reported that the device was moderately easy to use (mean score 3.1/5.0), that they would encourage others to use it (mean score 4.3/5.0), and that they liked the system overall (mean score 4.2/5.0). However, participants encountered some technical difficulties during use (mean score 2.2/5.0). Caregivers also found the device setup appropriate for the home environment and easy to use (mean score 3.5/5.0).

In people with MS, Dubuisson et al [91] reported that 66.7% of participants preferred the portable in-home 9HPT in comparison with the standard in-clinic version.

### Safety

Only 3% (2/74) of studies reported safety data [37,68]. Hoffman et al [68] reported that participants who received assessment via telerehabilitation were accompanied by a researcher to ensure safety. Boroojerdi et al [37] used a wearable patch and reported no adverse skin reactions at the application site or device malfunction. Adverse events were not reported in any of the included studies.

### Stage of Development and Implementation

As the assessments in this review were novel, the availability for clinical implementation varied. Most studies (44/74, 59%) evaluated assessments requiring specialized equipment for implementation. These devices included specialized cameras, wearable devices, electromyography, and specialized keyboards. Although not an application, the cardboard 9HPT developed by Dubuisson et al [91] was designed specifically to be environmentally friendly, cost-effective, and used by patients at home. The remaining external devices evaluated in this review were designated as developmental, with a need for subsequent safety and prospective studies on usability before clinical use.

Approximately 3% (2/74) of studies using telerehabilitation methods required videoconferencing devices and a stable internet connection for both providers and patients for implementation. However, although Hoffman et al [68] similarly used telerehabilitation methods, their protocol required participants to use clinical equipment during in-home assessments (eg, a hand dynamometer and the 9HPT), potentially limiting widespread implementation.

A smartphone or tablet-based application was used in 27% (20/74) of studies to administer assessments. The FLOODLIGHT application studied by Creagh et al [59] is currently available for download for iOS and Android devices. The remaining applications were study-specific developments but, given compatible devices and secure broadband internet connection availability, have limited barriers to implementation.

## Discussion

### Principal Findings

The purpose of this review was to systematically gather available literature on remote assessments for monitoring hand function in people with central, chronic, and neurological diseases. The search yielded 74 studies that met the inclusion criteria, and 71 unique assessments were examined for validity, reliability, and clinical implementation. A wide variety of metrics were collected on a number of hand function domains, including the amplitude of finger tapping, finger dexterity, hand tremor, and ADL independence. Altogether, the studies provide a number of insights; however, to date, no single tool, or combination of tools, validly and reliably captures hand function across these major neurological conditions.

Many of the studies were of good quality, and several study characteristics were found to enhance their quality. Including controls with nonneurological conditions as a comparison, when available, helped demonstrate the discriminant validity of the novel assessments examined. Most studies included participants with lower disability status, which likely allowed for more dynamic testing of hand function domains. Unfortunately, most of the included studies reported statistically insignificant associations with standard in-clinic metrics. As prior literature suggests that traditional in-clinic assessments have limited granularity for upper limb function in populations with neurological conditions, differences between the novel assessments and these traditional in-clinic tests could indicate that the new tools capture additional aspects of function (eg, quantifying pincer grasp) relative to the traditional in-clinic assessments or vice versa. In addition, few studies reported reliability, especially interrater reliability, suggesting the need for more research and that the included tools remain primarily in the development phase.

The most commonly assessed hand function domain was finger tapping speed, with moderate to high agreement across comparison assessments. The finger tapping test is a valid and reliable measure of bradykinesia in PD [94] and a predictor of ADL independence in acute stroke [95]. It is relatively simple to quantify finger tapping in-clinic or via a smartphone or tablet app by counting the number of finger taps within a specific time frame. Although overall construct validity and participant satisfaction were high, further work in other hand function domains will help determine the most salient predictors of ADL independence and response to treatment and intervention.

This review highlights important aspects of the feasibility of remote evaluations. Participant and caregiver satisfaction, when reported, were moderate to high for these technologically innovative assessments. This suggests that participants found the novel assessments easy to use and effective in evaluating their hand function despite being nontraditional. Further, 28% (21/74) of the included studies demonstrated the feasibility of remotely monitoring hand function over multiple days. This is a key finding, as long-term monitoring of hand function in a patient’s natural environment has the potential to identify changes in real time, allowing for timely intervention modifications.

Regarding patient safety, although the included assessments were noninvasive and posed a relatively low safety risk, ensuring the secure transfer of data, especially with internet-based communication (eg, telerehabilitation and smartphone or tablet-based apps) between patient and clinician, is critical to confidentiality and Health Insurance Portability Accountability Act compliance. Future studies should report on data storage and encryption methodologies.

The assessments evaluated were in varying stages of development and implementation. The most readily implementable types of assessment were those using telerehabilitation or smartphone- or tablet-based apps. According to 2019 data, 85% of Americans own a smartphone, and 93% use the internet regularly, of whom 75% use a home high-speed broadband network [96]. Given these statistics, telerehabilitation and application-based assessments, if interoperable across devices, might be relatively accessible for most patients. Lower costs could make clinical implementation less of a challenge. Furthermore, with no specialized devices to purchase or distribute to patients, clinics could similarly benefit from these cost-effective measures.

### Limitations

A major limitation of this review is the heterogeneity of hand function domains evaluated, which, when compounded with the methodological variability (in comparison assessments, inclusion criteria, and statistical approaches), made it difficult to compare the various tools. Future studies that include more homogeneous patient populations and standardized reporting of correlation coefficients with comparison assessments will facilitate analysis across domains and assessment types. A second limitation was the paucity of studies conducting repeated trials of the assessments, limiting the identification of any practice effects with use of a new device. In repeated trials of smartphone-based assessments, performance improved in the first 10 trials because of a practice effect, followed by a narrowing of variance as the practice effect waned and familiarity with the assessment increased [97]. Follow-up studies should include repeated trials, preferably over multiple days, to capture these effects and fluctuations in disease progression. Third, the effect of confounding variables (eg, disease-modifying therapies, age, and disease duration) was infrequently described in validity statistics; the generalizability of this review should proceed with caution. Fourth, all tools included require active participant engagement as opposed to passive monitoring (eg, collecting data on dexterity as a participant types to complete a survey). Passive monitoring may be able to capture similar metrics with a reduced participant time burden. Finally, we may have missed relevant studies published in non-English languages.

### Conclusions

This review suggests that remote assessments can be valid and reliable tools for measuring hand function impairments in chronic neurological diseases and that doing so is clinically feasible and acceptable to patients. In the past decade, personal smartphone and computer ownership have become commonplace; with it, patients and health care providers are able to communicate in real time, opening new avenues for care delivery and disease monitoring. We highlight the current potential to implement remote assessments via telerehabilitation and smartphone- or tablet-based apps. As interventions for ambulation and lower extremity function become increasingly robust, these methods will allow clinicians to reliably assess multiple domains of hand function to monitor disease progression and response to interventions.

## Appendix

Multimedia Appendix 1 Summary of studies.

Multimedia Appendix 2 Quality assessment of studies.

## Abbreviations

9HPT: 9-hole peg test
ADL: activities of daily living
ARAT: Action Research Arm Test
CNS: central nervous system
IADL: instrumental activities of daily living
MDS-UPDRS: Movement Disorder Society–Unified Parkinson’s Disease Rating Scale
MS: multiple sclerosis
PD: Parkinson disease
PRISMA: Preferred Reporting Items for Systematic Reviews and Meta-Analyses
