# Supplementary material for: Remote Assessments of Hand Function in Neurological Disorders: Systematic Review
Source: JMIR Rehabil Assist Technol. 2022 Mar 9;9(1):e33157. doi: 10.2196/33157 (PMC8943610; doi:10.2196/33157)
Supplement: Multimedia Appendix 1 [file rehab_v9i1e33157_app1.docx]

| Supplementary Table 1. Summary of Studies | | | |  |  |  |  | N | | Age [mean (SD)] | | Sex | |
| --- | --- | --- | --- | --- | --- | --- | --- | --- | --- | --- | --- | --- | --- |
| Author/Year | Study Design (duration) | | Diagnosis/ phenotype | Disease-specific severity levels | Modality of Assessment | Assessment Name | Hand function domains | Control | Experimental | Control | Experimental | Control | Experimental |
| Adams (2018) | Longitudinal (8 months) | | PD |  | Smartphone |  | Hand tremor | 128 | 337 |  |  |  |  |
| Aghanavesi (2017) | Cross-Sectional | | PD | Mean H&Y: 3.16 | External Device, Keyboard |  | Finger tapping Handwriting | 22 | 19 | 64.2 (7.4) | 71.4 (6.3) | 14M, 5F | 16M, 6F |
| Akram (2020) | Cross-Sectional | | PD |  | Smartphone |  | Finger tapping | 24 | 45 | 66.0 (11.8) | 61.9 (7.25) | 8M, 16F | 26M, 19F |
| Albani (2019) | Longitudinal  (12 weeks) | | PD | Mean H%Y: 2.50 | External Device, Sensors |  | Finger tapping Whole hand grasp | 15 | 25 | 66.4 | 66.9 | 9M, 6F | 15 M, 10 F |
| Amano (2018) | Cross-sectional | | Stroke | Median Brunnstrom Stage: 5.0 | Telerehabilitation |  | Finger dexterity Whole hand grasp Pincer grasp |  | 30 |  | 65.5 |  | 18M, 12F |
| Arora (2015) | Longitudinal  (34.4 days) | | PD | Mean UPDRS: 19.6 | Smartphone |  | Finger tapping | 10 | 10 | 57.7 (14.3) | 65.1 (9.8) | 40M, 6F | 7M, 3F |
| Arroyo-Gallego (2017) | Cross-Sectional | | PD | Mean UPDRS: 17.8 | Smartphone |  | Finger tapping | 27 | 24 | 54.35 (13.95) | 59.24 (11.43) | 4M, 19F | 10M, 11F |
| Bazgir (2018) | Cross-Sectional | | PD |  | External Device, Sensors |  | Hand tremor |  | 36 |  | 54 (13) |  | 20M, 16F |
| Bochniewicz (2017) | Cross-sectional | | Stroke | Mean ARAT score: 3.5 | External Device, Sensors |  | IADLs | 10 | 10 | 43 ± 15.9 | 56 ± 10.4 | 4M, 6F | 8M, 2F |
| Boroojerdi (2019) | Longitudinal  (3 days) | | PD | Mean H&Y: 2.2 | External Device, Sensors | NIMBLE patch | Finger tapping Hand tremor |  | 25 |  | 64.2 (7.8) |  | 15M, 10F |
| Burdea (2020) | Longitudinal  (4 weeks) | | Stroke |  | External Device, Virtual reality game | BrightBrainer system | Pincer grasp |  | 7 |  | 64.1 |  | 4 M, 3F |
| Cabrera-Martos (2019) | Cross-sectional | | PD | Mean UPDRS: 22.0 | Telerehabilitation |  | Finger dexterity Finger tapping Hand tremor |  | 21 |  | 70.9 ± 9.6 |  | 11M, 10F |
| Cai (2018) | Cross-Sectional | | PD |  | External Device, Sensors | A-WEAR bracelet | Hand tremor | 14 | 34 | 61.6 (12.8) | 64.0 (11.3) | 10M, 4F | 27M, 7F |
| Channa (2021) | Cross-Sectional | | PD | Mean UPDRS: 18.91 | External Device, Sensor |  | Hand tremor | 20 | 20 | 71.65 (6.87) | 70.25 (6.31) | 16M, 4F | 5M, 15F |
| Cole (2014) | Cross-Sectional | | PD |  | External Device, Sensor | Visualbasic | Hand tremor | 4 | 8 | 54 (16.6) | 62.9 (5.3) | 4M, 0F | 7M, 1F |
| Creagh (2020) | Cross-sectional | | MS | Median EDSS: 2.1 | Smartphone | FLOODLIGHT | Handwriting | 22 | 21 | 34 ± 9 | 40 ± 8 | 15M, 7F | 49M, 22F |
| Cunningham (2011) | Longitudinal (4 days) | | PD | Mean UPDRS: 53.9 | External Device, Keyboard | electromagnetic tracking system | Hand tremor |  | 10 |  | 68.2 (5.2) |  | 9M, 1F |
| Dai (2021) | Cross-Sectional | | PD |  | External Device/Smartphone | SENSE-PARK | Finger tapping  Hand tremor | 30 | 45 | 61.35 (6.56) | 64.53 (9.31) | 20M, 10F | 35M, 10F |
| Dubuisson (2017) | Cross-sectional | | MS |  | External Device |  | Finger dexterity | 68 | 109 | 41 ± 16.12 | 49 ± 12.5 | 18M, 50F | 38M, 71 F |
| Ferreira (2015) | Longitudinal (12 weeks) | | PD | Median UPDRS: 40 | External Device, Keyboard | neuroQWERTY | Hand tremor |  | 22 |  | 60.2 (9.8) |  | 14M, 8F |
| Giancardo (2016) | Cross-Sectional | | PD | Mean UPDRS: 20.6 | External Device, Keyboard | Kinesia | Finger tapping | 43 | 42 | 60.1 (10.2) | 59.0 (9.8) | 17M, 26F | 24M, 18F |
| Giuffrida (2009) | Cross-Sectional | | PD |  | External Device, Sensor |  | Hand tremor |  | 60 |  |  |  |  |
| Goetz (2009) | Longitudinal (6 months) | | PD | Mean UPDRS: 19.5 | External Device, Sensor |  | Finger tapping  Hand tremor |  | 52 |  | 63.8 (8.9) |  | 32M, 20F |
| Halloran (2016) | Longitudinal (8 weeks) | | Stroke |  | External Device, Sensor | MOX5 | ADLs |  | 24 |  | 61.8 (14.3) |  | 18M, 6F |
| Heijmans (2019) | Case study | | PD |  | External Device, Sensor |  | Hand tremor |  | 1 |  | 65 |  | 1M |
| Hoffman (2008) | Cross-sectional | | PD |  | Telerehabilitation |  | Finger dexterity Grip strength Hand tremor Handwriting Pinch strength | 6 | 6 |  | 66.1 (8.5) |  | 6M, 6F |
| Hssayeni (2019) | Cross-Sectional | | PD | Mean UPDRS: 21.8 | Smartphone |  | Hand tremor |  | 24 |  | 58.9 (9.3) | 14M, 10F | 14M, 10F |
| Iakovakis (2018) | Cross-Sectional | | PD | Mean UPDRS: 16.8 | Smartphone | iPrognosis | Finger tapping | 13 | 18 | 57 (3.9) | 61 (8.4) | 8M, 7F | 14M, 4F |
| Iakovakis (2020) | Cross-Sectional | | PD | Mean UPDRS: 19.7 | External Device, Keyboard |  | Finger tapping | 17 | 22 | 54.6 (9.4) | 58.6 (8.4) | 10M, 7F | 16M, 6F |
| Jeon (2017) | Cross-Sectional | | PD |  | Smartphone | CloudUPDRS | Hand tremor |  | 85 |  | 65.9 (9.2) |  | 41M, 44F |
| Jha (2020) | Crossover-Randomized | | PD | Median H&Y: 2 | External Device, Sensor | SNUMAP | Finger tapping  Hand tremor |  | 62 | 68 (median) |  | 42M, 20F |  |
| Kim (2018) | Cross-Sectional | | PD | Mean H&Y: 2.3 | External Device | Myo armband | Hand tremor |  | 92 |  | 67.1 (9.0) |  | 45M, 47F |
| Kleinholdermann (2021) | Cross-Sectional | | PD | Mean H&Y: 2.6 | External Device, Sensors | Neurokeys | Finger tapping |  | 45 |  | 59.2 (8.9) |  | 34M, 11F |
| Kostikis (2015) | Cross-sectional | | PD |  | Smartphone |  | Hand tremor | 20 | 25 | 67.2 (6.3) | 70.9 (11.8) | 10M, 10F | PD: 11M, 12F PD de novo (before meds): 1M, 1F |
| Lam (2020) | Cross-Sectional | | MS | Median EDSS: 2.5 | Smartphone | smartphone tapper (SmT) | Finger dexterity | 24 | 102 | 45.2 (13.5) | 46.4 (10.1) | 8M, 10F | 21M, 64F |
| Lee, C (2016) | Cross-Sectional | | PD | Mean UPDSR: 20.0 | External Device, Virtual reality | Kinect v2 | Finger tapping | 87 | 57 | 53.4 (14.8) | 65.4 (9.0) | 34M, 53F | 34M, 23F |
| Lee, S (2018) | Cross-Sectional | | Stroke |  | Smartphone | HLTapper | Whole hand grasp |  | 10 |  | 58 (16.5) |  | 6M, 4F |
| Lee, U (2016) | Cross-Sectional | | PD |  | External Device, Sensors |  | Finger tapping | 11 | 9 | 83.8 | 85.8 | 3M, 8F | 1M, 8F |
| Lin (2019) | Cross-Sectional | | Stroke |  | Smartphone | mPower | Finger dexterity Whole hand grasp |  | 15 |  | 59.6 (16.3) |  | 9M, 6F |
| Lipsmeier (2018) | Longitudinal (6 months) | | PD | Man UPDRS: 45.41 | External Device, Smartwatch | Smartwatch3 | Finger tapping  Hand tremor | 35 | 43 | 56.23 (7.83) | 57.5 (8.45) | 27M, 8F | 35M, 8F |
| Londral (2016) | Cross-sectional | | ALS |  | External Device, Keyboard |  | Finger dexterity | 26 | 19 |  | 64 (median) |  | 3M, 16F |
| Lopez-Blanco (2019) | Longitudinal (12 months) | | PD | Mean UPDRS: 19 | External Device, Smartwatch | APDM, Biostamp | Hand tremor |  | 22 |  | 72 (7.6) |  | 13M, 9F |
| Mahadevan (2020) | Cross-Sectional | | PD |  | External Device, Sensors |  | Hand tremor | 50 | 31 | 43.9 (10.02) | 68.1 (8.13) | 23M, 27F | 20M, 11F |
| Matarazzo (2019) | Longitudinal (6 months) | | PD | Median H&Y: 2 | External Device, Keyboard | spiral drawing assessment | Finger tapping | 30 | 29 | 63.0 (56.48-69.44) | 59.78 (54.19-68.60) | 14M, 16F | 15M, 14F |
| Memedi (2015) | Longitudinal (3 years) | | PD | Mean UPDRS: 49 | External Device, Sensors |  | Handwriting | 10 | 65 | 61 (7) | 65 (11) | 5M, 5F | 43M, 22F |
| Mera (2012) | Cross-Sectional | | PD |  | External Device, Sensors | BRAIN | Finger tapping | 10 | 10 |  | 61.4 (7.4) | 8M, 2F | 8M, 2F |
| Mitsi (2017) | Cross-sectional | | PD | Mean UPDRS: 26.3 | Tablet | iMotor | Finger tapping Reaction time | 17 | 19 | 53.0 (17.3) | 67.8 (8.8) | 8M, 9F | 10M, 9F |
| Noyce (2014) | Cross-Sectional | | PD | Median H&Y: 2 | Smartphone, Keyboard | Apkinson | Finger tapping | 93 | 58 | 60.5 (13.1) | 63.0 (10.6) | 32M, 61F | 37M, 21F |
| Orozco-Arroyave (2020) | Cross-Sectional | | PD | Mean UPDRS: 37.05 | Smartphone | PD Dr | Finger tapping  Hand tremor | 60 | 23 | 62.2 (10.2) | 68.6 (11.3) | 30M, 30F | 11M, 12F |
| Pan (2015) | Cross-Sectional | | PD | Median H&Y: 2 | Smartphone |  | Hand tremor |  | 40 |  | 68.5 (9.5) |  | 35M, 5F |
| Papadopoulos (2021) | Cross-Sectional | | PD | Mean UPDRS: 19.7 | Smartphone |  | Hand tremor | 14 | 31 | 55.4 (11.7) | 62.1 (7.3) |  |  |
| Powers (2021) | Longitudinal (6 months) | | PD |  | External Device, Smartwatch |  | Hand tremor |  | 225 |  | 71.4 (8.9) |  | 156M, 69F |
| Pratap (2020) | Longitudinal (12 weeks) | | MS |  | Smartphone | SymptoMS Screen | Finger tapping | 134 | Self-referred:359 Confirmed: 136 | 36.9 (11.4) | Self-referred: 45.2 (11.6) Confirmed: 48.9 (11.2) | 15M, 27F | Self-referred: 56M, 154F Confirmed: 14M, 78F |
| Prochazka (2015) | Cross-sectional | | SCI |  | External Device, Sensors | Rejoice Arm and Hand Function Test (RAHFT) | Finger dexterity Whole hand grasp Pincer grasp |  | 13 |  | 24-56 |  |  |
| Rigas (2012) | Cross-Sectional | | PD |  | External Device, Sensors |  | Hand tremor | 5 | 18 | 63.9 (6.2) |  |  |  |
| Salarian (2007) | Cross-Sectional | | PD |  | External Device, Sensors | Kinesia | Hand tremor | 10 | 10 | 63.6 (10.5) | 61.5 (7.8) | 5M, 5F | 5M, 5F |
| San-Segundo (2020) | Cross-Sectional | | PD |  | External Device, Sensors |  | Hand tremor |  | 12 |  | 62-85 |  |  |
| Sanchez-Perez (2018) | Cross-Sectional | | PD |  | Tablet |  | Hand tremor |  | 57 |  | 66.4 (9.0) |  | 35M, 22F |
| Schallert (2020) | Cross-Sectional | | MS, PD, stroke, cerebellar ataxia |  | External Device, Tablet | BRAIN | Finger tapping Handwriting | 25 | 29 | 58.3 (16.1) | 46.6 (14.8) | 14M, 15F | 10M, 15F |
| Shribman (2017) | Cross-Sectional | | MS |  | External Device, Keyboard |  | Finger tapping |  | 39 |  | 43.2 |  | 10M, 29F |
| Sigcha (2021) | Longitudinal (8 weeks) | | PD |  | Smartphone | SMART | Hand tremor |  | 18 |  | 64.9 (7.6) |  | 8M, 10F |
| Simonet (2021) | Cross-Sectional | | PD | Mean UPDRS: 21.2 | External Device, Sensors |  | Finger tapping | 30 | 26 | 63.8 (7.2) | 59.6 (10.9) | 11M, 19F | 17M, 9F |
| Stamatakis (2013) | Cross-Sectional | | PD |  | External Device, Sensors |  | Finger tapping |  | 36 |  | 63.9 (9.1) |  | 28M, 8F |
| Tavares (2005) | Pre/Post-Interventional | | PD |  | External Device, Sensors |  | Finger tapping |  | 62 |  | 59.9 (8.8) |  |  |
| Trager (2020) | Cross-sectional | | PD | Mean UPDRS: 35.5 | External Device, Keyboard |  | Finger dexterity Finger tapping | 11 | 16 | 63.2 (6.6) | 68.9 (8.7) | 5M, 6F | 12 M, 4F |
| Westin (2010) | Longitudinal (1 week) | | PD | Mean UPDRS: 49.0 | Smartphone | iMotor | Handwriting |  | 60 |  | 64.9 (7.3) |  | 39M, 21F |
| Wissel (2018) | Cross-Sectional | | PD | Mean UPDRS: 48.6 | External Device, Tablet |  | Finger tapping | 11 | 11 | 62.5 (10.5) | 60.6 (9.0) | 5M, 6F | 8M, 3F |
| Wu (2020) | Cross-Sectional | | PD |  | External Device, Sensors |  | Hand tremor |  | 17 |  | 63.3 (6.6) |  | 11M, 6F |
| Yu (2016) | Longitudinal (12 weeks) | | Stroke | FMA: 18.3 | External Device, Sensors | Quantitative Fugl-Meyer | Finger dexterity Pinch strength | 24 |  |  | 69.4 (12.8) |  | 16 M, 8 F |
| Zambrana (2019) | Cross-Sectional | | Stroke |  | Smartphone | HopkinsPD | ADLs | 15 | 6 | 31.2 (4.6) | 55.3 (16.9) | 7M, 8F | 4M, 2F |
| Zhan (2016) | Longitudinal (6 months) | | PD |  | External Device, Sensors | Axivity AX3 | Finger tapping  Hand tremor | 105 | 121 | 45.4 (15.5) | 57.6 (9.1) | 56M, 49F | 71M, 50F |
| Zhang (2020) | Longitudinal (4 weeks) | | PD |  | External Device, Sensors |  | Hand tremor |  | 12 |  | 65-85 |  | 8M, 4F |
|  | | Legend PD: Parkinson’s disease; MS: multiple sclerosis; ALS: amyotrophic lateral sclerosis; H&Y: Hoen & Yahr; SCI: spinal cord injury; IADLs: instrumental activities of daily living; ADLs: activities of daily living; UPDRS: Unified Parkinson’s Disease Rating Scale; ARAT: Action Research Arm Test; FMA: Fugl-Meyer Assessment | | | | | | | | | | | |
