# Supplementary material for: Remote Assessments of Hand Function in Neurological Disorders: Systematic Review
Source: JMIR Rehabil Assist Technol. 2022 Mar 9;9(1):e33157. doi: 10.2196/33157 (PMC8943610; doi:10.2196/33157)
Supplement: Multimedia Appendix 2 [file rehab_v9i1e33157_app2.docx]

| Supplementary Table 2. Quality Assessment of Studies | | |  |  |  |  |  |  |  |
| --- | --- | --- | --- | --- | --- | --- | --- | --- | --- |
| Study (Year) | Was the research question/objective clearly stated? | Was the study population clearly specified? | Was a sample size justification or power description? | Were the exposure measures clearly defined, valid, reliable, and implemented consistently? | Was the exposure assessed more than once over time? | Were the outcome measures clearly defined, valid, reliable, and implemented consistently across? | Were the assessors blinded to the exposure status of participants? | Was loss to follow-up after baseline 20% or less? | Quality Rating (Good, Fair, or Poor) |
| Adams (2018) | Yes | No | No | Yes | Yes | No | No | No | Poor |
| Aghanavesi (2017) | Yes | Yes | No | Yes | Yes | Yes | No | NA | Good |
| Akram (2020) | Yes | Yes | No | Yes | Yes | Yes | No | Yes | Good |
| Albani (2019) | Yes | Yes | No | Yes | Yes | Yes | Yes | NR | Good |
| Amano (2018) | Yes | Yes | Yes | Yes | No | Yes | Yes | NA | Good |
| Arora (2015) | Yes | Yes | Yes | Yes | Yes | Yes | No | Yes | Good |
| Arroyo-Gallego (2017) | Yes | Yes | No | Yes | No | Yes | No | NA | Good |
| Bazgir (2018) | Yes | Yes | No | Yes | No | Yes | No | NA | Good |
| Bochniewicz (2017) | Yes | Yes | No | Yes | No | Yes | No | NA | Good |
| Boroojerdi (2019) | Yes | Yes | Yes | Yes | Yes | Yes | No | Yes | Good |
| Burdea (2020) | Yes | Yes | Yes | Yes | Yes | Yes | Yes | Yes | Good |
| Cabrera Martos (2019) | Yes | Yes | Yes | Yes | No | Yes | Yes | NA | Good |
| Cai (2018) | Yes | Yes | No | Yes | No | Yes | No | NA | Fair |
| Channa (2021) | Yes | Yes | No | Yes | Yes | Yes | No | No | Fair |
| Cole (2014) | Yes | Yes | No | Yes | No | No | No | NA | Poor |
| Creagh (2020) | Yes | Yes | No | Yes | No | Yes | No | NA | Fair |
| Cunningham (2011) | Yes | Yes | No | Yes | Yes | Yes | No | No | Fair |
| Dai (2021) | Yes | Yes | No | Yes | No | Yes | No | NA | Fair |
| Dubuisson (2017) | Yes | Yes | No | Yes | No | Yes | No | NA | Fair |
| Ferreira (2015) | Yes | Yes | No | Yes | Yes | Yes | No | No | Good |
| Giancardo (2016) | Yes | Yes | No | Yes | No | Yes | No | NA | Fair |
| Giuffrida (2009) | Yes | No | No | Yes | No | Yes | No | NA | Poor |
| Goetz (2009) | Yes | Yes | No | Yes | Yes | Yes | No | No | Fair |
| Halloran (2016) | Yes | No | No | Yes | Yes | No | No | NA | Fair |
| Heijmans (2019) | Yes | Yes | No | No | Yes | Yes | No | No | Poor |
| Hoffman et al (2008) | Yes | Yes | No | Yes | No | Yes | Yes | NA | Good |
| Hssayeni (2019) | Yes | Yes | No | Yes | No | Yes | No | NA | Good |
| Iakovakis (2018) | Yes | Yes | No | Yes | No | Yes | No | NA | Good |
| Iakovakis (2020) | Yes | No | No | Yes | No | Yes | No | NA | Poor |
| Jeon (2017) | Yes | Yes | No | Yes | No | Yes | No | NA | Fair |
| Jha (2020) | Yes | Yes | No | Yes | Yes | Yes | Yes | NA | Good |
| Kim (2018) | Yes | Yes | No | Yes | No | Yes | No | NA | Good |
| Kleinholdermann (2021) | Yes | Yes | No | Yes | No | Yes | Yes | NA | Good |
| Kostikis (2015) | Yes | Yes | No | Yes | No | Yes | No | NA | Good |
| Lam (2020) | Yes | Yes | No | Yes | No | Yes | No | Yes | Good |
| Lee, C (2016) | Yes | Yes | No | Yes | No | Yes | No | NA | Good |
| Lee, S (2018) | Yes | Yes | No | Yes | No | Yes | No | NA | Good |
| Lee, U (2016) | Yes | No | No | Yes | No | Yes | No | NA | Poor |
| Lin (2019) | Yes | Yes | No | Yes | No | Yes | No | NA | Good |
| Lipsmeier (2018) | Yes | Yes | No | Yes | No | Yes | No | NA | Good |
| Londral (2016) | Yes | Yes | No | Yes | No | Yes | Yes | NA | Good |
| Lopez-Blanco (2019) | Yes | Yes | No | Yes | Yes | Yes | Yes | Yes | Good |
| Mahadevan (2020) | Yes | Yes | No | Yes | No | Yes | Yes | NA | Good |
| Matarazzo (2019) | Yes | Yes | Yes | Yes | Yes | Yes | No | Yes | Good |
| Memedi (2015) | Yes | Yes | No | Yes | Yes | Yes | No | Yes | Good |
| Mera (2012) | Yes | No | No | Yes | Yes | Yes | No | Yes | Good |
| Mitsi et al (2017) | Yes | Yes | Yes | Yes | No | Yes | No | NA | Good |
| Noyce (2014) | Yes | Yes | No | Yes | No | Yes | No | NA | Good |
| Orozco-Arroyave (2019) | Yes | Yes | No | Yes | No | Yes | No | NA | Good |
| Pan (2015) | Yes | No | No | No | Yes | Yes | No | No | Poor |
| Papadopoulos (2021) | Yes | No | No | Yes | No | Yes | No | NA | Poor |
| Powers (2021) | Yes | Yes | No | Yes | Yes | Yes | Yes | Yes | Good |
| Pratap (2020) | Yes | Yes | No | Yes | Yes | Yes | No | Yes | Good |
| Prochazka (2015) | Yes | Yes | No | Yes | No | Yes | Yes | NA | Good |
| Rigas (2012) | Yes | Yes | No | Yes | No | Yes | No | NA | Good |
| Salarian (2007) | Yes | Yes | No | Yes | No | Yes | No | NA | Good |
| San-Segundo (2020) | Yes | No | No | Yes | Yes | Yes | No | Yes | Fair |
| Sanchez-Perez (2018) | Yes | Yes | No | Yes | No | Yes | No | NA | Good |
| Schallert (2020) | Yes | Yes | No | Yes | No | Yes | Yes | Yes | Good |
| Shribman (2017) | Yes | Yes | No | Yes | No | Yes | No | NA | Good |
| Sigcha (2021) | Yes | Yes | No | Yes | Yes | Yes | No | NA | Good |
| Simonet (2021) | Yes | Yes | No | Yes | Yes | Yes | No | NA | Good |
| Stamatakis (2013) | Yes | Yes | No | Yes | No | Yes | No | NA | Good |
| Tavares (2005) | Yes | Yes | No | Yes | Yes | Yes | No | Yes | Good |
| Trager (2020) | Yes | Yes | No | Yes | No | Yes | No | NA | Good |
| Westin (2010) | Yes | Yes | No | Yes | Yes | Yes | Yes | No | Good |
| Wissel (2018) | Yes | Yes | Yes | Yes | No | Yes | Yes | NA | Good |
| Wu (2020) | Yes | Yes | No | Yes | No | No | No | NA | Fair |
| Yu (2016) | Yes | Yes | No | Yes | Yes | Yes | No | Yes | Good |
| Zambrana (2019) | Yes | Yes | No | Yes | No | Yes | No | NA | Fair |
| Zhan (2016) | Yes | Yes | No | Yes | Yes | Yes | No | No | Fair |
| Zhang (2020) | Yes | Yes | No | No | No | Yes | No | NA | Poor |
